# Supplementary material for: Modeling glioblastoma heterogeneity as a dynamic network of cell states
Source: Mol Syst Biol. 2021 Sep 16;17(9):e10105. doi: 10.15252/msb.202010105 (PMC8444284; doi:10.15252/msb.202010105)
Supplement: Supplementary file 5 — Source Data for Figure 3 [file MSB-17-e10105-s001.zip › Figure3A_sourcedata/GSEA_3065/nosigdb_state1.GseaPreranked.1622718409535/gsea_report_for_na_neg_1622718409535.html]

Report for na\_neg 1622718409535 [GSEA]

| GS  follow link to MSigDB | GS DETAILS | SIZE | ES | NES | NOM p-val | FDR q-val | FWER p-val | RANK AT MAX | LEADING EDGE || 1 | LARSSON\_STATE\_2 | Details ... | 85 | -0.95 | -3.25 | 0.000 | 0.000 | 0.000 | 543 | tags=98%, list=5%, signal=102% |
| 2 | LARSSON\_STATE\_3 | Details ... | 86 | -0.86 | -2.99 | 0.000 | 0.000 | 0.000 | 787 | tags=78%, list=8%, signal=84% |
| 3 | DOROSZKO\_U3180MG\_CLUSTER\_2 | Details ... | 465 | -0.71 | -2.96 | 0.000 | 0.000 | 0.000 | 1410 | tags=57%, list=14%, signal=64% |
| 4 | RICHARDS\_NOWAKOWSKI\_SCIENCE\_2017\_OPC\_UPREG | Details ... | 324 | -0.69 | -2.81 | 0.000 | 0.000 | 0.000 | 1158 | tags=51%, list=12%, signal=55% |
| 5 | RICHARDS\_ZHONG\_NATURE\_2018\_OPC\_UPREG | Details ... | 72 | -0.81 | -2.67 | 0.000 | 0.000 | 0.000 | 927 | tags=68%, list=9%, signal=75% |
| 6 | RICHARDS\_NOWAKOWSKI\_SCIENCE\_2017\_ASTROCYTE\_UPREG | Details ... | 487 | -0.64 | -2.67 | 0.000 | 0.000 | 0.000 | 1028 | tags=42%, list=10%, signal=45% |
| 7 | GAROFANO\_IAVARONE\_NEU\_SUBTYPE | Details ... | 169 | -0.69 | -2.57 | 0.000 | 0.000 | 0.000 | 2173 | tags=61%, list=22%, signal=77% |
| 8 | IVY\_CT | Details ... | 145 | -0.67 | -2.52 | 0.000 | 0.000 | 0.000 | 845 | tags=40%, list=9%, signal=43% |
| 9 | SINGH\_SOX2\_UPREGULATED\_TRANSCRIPTS\_PMID\_28122245 | Details ... | 429 | -0.60 | -2.49 | 0.000 | 0.000 | 0.000 | 1131 | tags=30%, list=11%, signal=32% |
| 10 | RICHARDS\_ZHONG\_NATURE\_2018\_ASTROCYTES\_UPREG | Details ... | 236 | -0.63 | -2.46 | 0.000 | 0.000 | 0.000 | 1163 | tags=47%, list=12%, signal=52% |
| 11 | RICHARDS\_VERHAAK\_CANCERCELL\_2010\_PRONEURAL | Details ... | 114 | -0.68 | -2.44 | 0.000 | 0.000 | 0.000 | 503 | tags=33%, list=5%, signal=35% |
| 12 | LARSSON\_STATE\_6 | Details ... | 101 | -0.68 | -2.39 | 0.000 | 0.000 | 0.000 | 949 | tags=45%, list=10%, signal=49% |
| 13 | SUVA\_AC\_PMID\_31327527 | Details ... | 32 | -0.82 | -2.38 | 0.000 | 0.000 | 0.000 | 489 | tags=69%, list=5%, signal=72% |
| 14 | OUTERRADIALGLIA | Details ... | 50 | -0.75 | -2.38 | 0.000 | 0.000 | 0.000 | 489 | tags=42%, list=5%, signal=44% |
| 15 | SUVA\_OPC\_PMID\_31327527 | Details ... | 37 | -0.80 | -2.37 | 0.000 | 0.000 | 0.000 | 944 | tags=62%, list=10%, signal=68% |
| 16 | POLLEN\_OUTERRADIALGLIA\_PMID\_26406371 | Details ... | 50 | -0.75 | -2.34 | 0.000 | 0.000 | 0.000 | 489 | tags=42%, list=5%, signal=44% |
| 17 | RICHARDS\_NEFTEL\_CELL\_2019\_AC | Details ... | 32 | -0.82 | -2.33 | 0.000 | 0.000 | 0.000 | 489 | tags=69%, list=5%, signal=72% |
| 18 | RICHARDS\_NEFTEL\_CELL\_2019\_OPC | Details ... | 37 | -0.80 | -2.33 | 0.000 | 0.000 | 0.000 | 944 | tags=62%, list=10%, signal=68% |
| 19 | TISIG\_PMID\_32649888 | Details ... | 34 | -0.78 | -2.28 | 0.000 | 0.000 | 0.000 | 821 | tags=50%, list=8%, signal=54% |
| 20 | RICHARDS\_NOWAKOWSKI\_SCIENCE\_2017\_VRG\_UPREG | Details ... | 392 | -0.55 | -2.27 | 0.000 | 0.000 | 0.000 | 1262 | tags=39%, list=13%, signal=42% |
| 21 | NB\_KI\_CLUSTER2 |  | 258 | -0.57 | -2.26 | 0.000 | 0.000 | 0.000 | 1770 | tags=45%, list=18%, signal=53% |
| 22 | DOROSZKO\_U3054MG\_CLUSTER\_0 |  | 61 | -0.70 | -2.24 | 0.000 | 0.000 | 0.000 | 1132 | tags=54%, list=11%, signal=61% |
| 23 | ALLEN\_114\_OPCL1\_6MYT1 |  | 16 | -0.89 | -2.19 | 0.000 | 0.000 | 0.000 | 282 | tags=69%, list=3%, signal=71% |
| 24 | DOROSZKO\_U3054MG\_CLUSTER\_5 |  | 105 | -0.61 | -2.18 | 0.000 | 0.000 | 0.000 | 1413 | tags=43%, list=14%, signal=49% |
| 25 | ALLEN\_112\_ASTROL1FGFR3MT1G |  | 25 | -0.79 | -2.15 | 0.000 | 0.000 | 0.000 | 652 | tags=64%, list=7%, signal=68% |
| 26 | SUVA\_NPC1\_PMID\_31327527 |  | 34 | -0.72 | -2.12 | 0.000 | 0.000 | 0.001 | 577 | tags=47%, list=6%, signal=50% |
| 27 | ALLEN\_113\_ASTROL1FGFR3FOS |  | 25 | -0.78 | -2.10 | 0.000 | 0.000 | 0.001 | 652 | tags=60%, list=7%, signal=64% |
| 28 | RICHARDS\_NEFTEL\_CELL\_2019\_NPC1 |  | 34 | -0.72 | -2.10 | 0.000 | 0.000 | 0.001 | 577 | tags=47%, list=6%, signal=50% |
| 29 | ALLEN\_111\_ASTROL1\_6FGFR3ETNPPL |  | 23 | -0.77 | -2.09 | 0.000 | 0.000 | 0.001 | 652 | tags=52%, list=7%, signal=56% |
| 30 | RICHARDS\_NOWAKOWSKI\_SCIENCE\_2017\_MGE\_RG1\_UPREG |  | 325 | -0.51 | -2.07 | 0.000 | 0.000 | 0.001 | 1483 | tags=38%, list=15%, signal=43% |
| 31 | GANDAL\_BIPOLAR\_UP\_PMID29439242 |  | 63 | -0.59 | -1.97 | 0.000 | 0.000 | 0.007 | 665 | tags=33%, list=7%, signal=36% |
| 32 | RICHARDS\_NOWAKOWSKI\_SCIENCE\_2017\_NIN1\_UPREG |  | 154 | -0.51 | -1.91 | 0.000 | 0.001 | 0.016 | 1436 | tags=36%, list=15%, signal=42% |
| 33 | NB\_KI\_CLUSTER8 |  | 313 | -0.47 | -1.89 | 0.000 | 0.001 | 0.020 | 2397 | tags=51%, list=24%, signal=65% |
| 34 | TIISIG\_PMID\_32649888 |  | 25 | -0.69 | -1.89 | 0.000 | 0.001 | 0.022 | 495 | tags=36%, list=5%, signal=38% |
| 35 | RICHARDS\_NOWAKOWSKI\_SCIENCE\_2017\_IPC\_NEN1\_UPREG |  | 233 | -0.47 | -1.85 | 0.000 | 0.001 | 0.039 | 1631 | tags=44%, list=17%, signal=52% |
| 36 | DOROSZKO\_U3054MG\_CLUSTER\_7 |  | 30 | -0.64 | -1.82 | 0.004 | 0.002 | 0.053 | 1679 | tags=40%, list=17%, signal=48% |
| 37 | RICHARDS\_VERHAAK\_CANCERCELL\_2010\_CLASSICAL |  | 120 | -0.50 | -1.79 | 0.000 | 0.002 | 0.074 | 1547 | tags=38%, list=16%, signal=44% |
| 38 | WANG\_CLASSICAL\_PMID\_28697342 |  | 37 | -0.61 | -1.79 | 0.004 | 0.002 | 0.074 | 921 | tags=38%, list=9%, signal=42% |
| 39 | GANDAL\_SCHIZOPHRENIA\_UP\_PMID29439242 |  | 80 | -0.52 | -1.78 | 0.002 | 0.003 | 0.089 | 712 | tags=31%, list=7%, signal=33% |
| 40 | NB\_KI\_CLUSTER23 |  | 255 | -0.44 | -1.74 | 0.000 | 0.004 | 0.120 | 1289 | tags=30%, list=13%, signal=33% |
| 41 | RICHARDS\_NOWAKOWSKI\_SCIENCE\_2017\_NEN\_EARLY2\_UPREG |  | 402 | -0.42 | -1.74 | 0.000 | 0.004 | 0.125 | 2174 | tags=44%, list=22%, signal=54% |
| 42 | GANDAL\_ALCOHOLADDICTION\_DN\_PMID29439242 |  | 60 | -0.53 | -1.72 | 0.000 | 0.004 | 0.147 | 1321 | tags=33%, list=13%, signal=38% |
| 43 | NB\_KI\_CLUSTER6 |  | 482 | -0.41 | -1.70 | 0.000 | 0.006 | 0.196 | 2502 | tags=44%, list=25%, signal=55% |
| 44 | RICHARDS\_NOWAKOWSKI\_SCIENCE\_2017\_NIN5\_UPREG |  | 264 | -0.43 | -1.70 | 0.000 | 0.006 | 0.202 | 1660 | tags=33%, list=17%, signal=38% |
| 45 | LARSSON\_STATE\_5 |  | 99 | -0.48 | -1.67 | 0.004 | 0.008 | 0.273 | 702 | tags=39%, list=7%, signal=42% |
| 46 | NB\_KI\_CLUSTER17 |  | 132 | -0.45 | -1.65 | 0.002 | 0.009 | 0.302 | 784 | tags=30%, list=8%, signal=32% |
| 47 | OLSEN\_SCPLIKE |  | 80 | -0.49 | -1.64 | 0.002 | 0.010 | 0.347 | 395 | tags=34%, list=4%, signal=35% |
| 48 | DOROSZKO\_U3013MG\_CLUSTER\_3 |  | 103 | -0.44 | -1.56 | 0.002 | 0.021 | 0.590 | 594 | tags=23%, list=6%, signal=25% |
| 49 | DOROSZKO\_U3013MG\_CLUSTER\_0 |  | 28 | -0.55 | -1.54 | 0.031 | 0.024 | 0.652 | 961 | tags=50%, list=10%, signal=55% |
| 50 | ALLEN\_116\_OLIGOL4\_6OPALIN |  | 21 | -0.59 | -1.54 | 0.029 | 0.024 | 0.663 | 1633 | tags=62%, list=17%, signal=74% |
| 51 | KRONA\_ROSEN\_WHITE\_MATTER\_INVASION |  | 200 | -0.39 | -1.53 | 0.000 | 0.027 | 0.717 | 1932 | tags=34%, list=20%, signal=41% |
| 52 | NB\_KI\_CLUSTER14 |  | 235 | -0.39 | -1.52 | 0.000 | 0.028 | 0.732 | 1843 | tags=31%, list=19%, signal=37% |
| 53 | RICHARDS\_NOWAKOWSKI\_SCIENCE\_2017\_NIN3\_UPREG |  | 146 | -0.40 | -1.52 | 0.011 | 0.028 | 0.740 | 626 | tags=18%, list=6%, signal=19% |
| 54 | RICHARDS\_NOWAKOWSKI\_SCIENCE\_2017\_IN\_CTX\_CGE2\_UPREG |  | 230 | -0.38 | -1.51 | 0.002 | 0.029 | 0.747 | 1123 | tags=25%, list=11%, signal=27% |
| 55 | VANGRONINGEN\_MES |  | 403 | -0.36 | -1.50 | 0.000 | 0.030 | 0.779 | 1363 | tags=29%, list=14%, signal=33% |
| 56 | WENG\_STEMNESS\_PMID\_30982771 |  | 37 | -0.51 | -1.50 | 0.029 | 0.031 | 0.782 | 1317 | tags=38%, list=13%, signal=43% |
| 57 | RICHARDS\_NOWAKOWSKI\_SCIENCE\_2017\_IN\_STR\_UPREG |  | 177 | -0.39 | -1.48 | 0.004 | 0.035 | 0.824 | 1660 | tags=31%, list=17%, signal=36% |
| 58 | DOROSZKO\_PERIVASCULAR\_INVASION |  | 35 | -0.50 | -1.45 | 0.046 | 0.042 | 0.885 | 391 | tags=37%, list=4%, signal=39% |
| 59 | ALMSTEDT\_ALK |  | 56 | -0.46 | -1.45 | 0.031 | 0.042 | 0.891 | 1321 | tags=27%, list=13%, signal=31% |
| 60 | RICHARDS\_LIDDELOW\_NATURE\_2017\_A1\_REACTIVE\_ASTROCYTES |  | 21 | -0.54 | -1.43 | 0.068 | 0.048 | 0.928 | 238 | tags=14%, list=2%, signal=15% |
| 61 | DOROSZKO\_U3054MG\_CLUSTER\_2 |  | 259 | -0.35 | -1.40 | 0.007 | 0.059 | 0.967 | 1288 | tags=27%, list=13%, signal=30% |
| 62 | RICHARDS\_NOWAKOWSKI\_SCIENCE\_2017\_IN\_CTX\_MGE2\_UPREG |  | 201 | -0.36 | -1.38 | 0.005 | 0.064 | 0.977 | 907 | tags=22%, list=9%, signal=24% |
| 63 | DEPRETER\_LOW\_NB\_RISK |  | 19 | -0.53 | -1.38 | 0.098 | 0.067 | 0.981 | 773 | tags=42%, list=8%, signal=46% |
| 64 | NB\_KI\_CLUSTER4 |  | 94 | -0.39 | -1.36 | 0.038 | 0.077 | 0.990 | 1129 | tags=31%, list=11%, signal=34% |
| 65 | RICHARDS\_NEFTEL\_CELL\_2019\_NPC2 |  | 32 | -0.46 | -1.33 | 0.094 | 0.091 | 0.997 | 339 | tags=25%, list=3%, signal=26% |
| 66 | RICHARDS\_ZHONG\_NATURE\_2018\_MICROGLIA\_UPREG |  | 215 | -0.34 | -1.32 | 0.017 | 0.095 | 0.998 | 915 | tags=22%, list=9%, signal=24% |
| 67 | RICHARDS\_NOWAKOWSKI\_SCIENCE\_2017\_IN\_CTX\_MGE1\_UPREG |  | 283 | -0.33 | -1.32 | 0.017 | 0.095 | 0.998 | 1616 | tags=28%, list=16%, signal=32% |
| 68 | SUVA\_NPC2\_PMID\_31327527 |  | 32 | -0.46 | -1.32 | 0.111 | 0.094 | 0.998 | 339 | tags=25%, list=3%, signal=26% |
| 69 | GAROFANO\_IAVARONE\_MTC\_SUBTYPE |  | 230 | -0.33 | -1.29 | 0.039 | 0.115 | 1.000 | 1583 | tags=20%, list=16%, signal=24% |
| 70 | ALLEN\_115\_OLIGOL4\_6MOBPCOL18A1 |  | 24 | -0.47 | -1.23 | 0.185 | 0.164 | 1.000 | 1245 | tags=46%, list=13%, signal=52% |
| 71 | DOROSZKO\_U3054MG\_CLUSTER\_1 |  | 16 | -0.49 | -1.23 | 0.212 | 0.163 | 1.000 | 213 | tags=25%, list=2%, signal=26% |
| 72 | RICHARDS\_NOWAKOWSKI\_SCIENCE\_2017\_MGE\_IPC3\_UPREG |  | 120 | -0.34 | -1.22 | 0.111 | 0.174 | 1.000 | 479 | tags=18%, list=5%, signal=18% |
| 73 | RICHARDS\_NOWAKOWSKI\_SCIENCE\_2017\_NIN4\_UPREG |  | 135 | -0.33 | -1.21 | 0.113 | 0.183 | 1.000 | 1307 | tags=24%, list=13%, signal=28% |
| 74 | IVY\_IT |  | 37 | -0.41 | -1.21 | 0.184 | 0.184 | 1.000 | 415 | tags=19%, list=4%, signal=20% |
| 75 | DOROSZKO\_U3054MG\_CLUSTER\_3 |  | 210 | -0.31 | -1.20 | 0.087 | 0.189 | 1.000 | 1559 | tags=25%, list=16%, signal=29% |
| 76 | RICHARDS\_NOWAKOWSKI\_SCIENCE\_2017\_IN\_CTX\_CGE1\_UPREG |  | 299 | -0.30 | -1.20 | 0.070 | 0.188 | 1.000 | 1618 | tags=24%, list=16%, signal=28% |
| 77 | NB\_KI\_CLUSTER7 |  | 324 | -0.29 | -1.19 | 0.076 | 0.202 | 1.000 | 4117 | tags=46%, list=42%, signal=76% |
| 78 | DOROSZKO\_U3054MG\_CLUSTER\_10 |  | 256 | -0.30 | -1.18 | 0.101 | 0.211 | 1.000 | 1102 | tags=19%, list=11%, signal=21% |
| 79 | RICHARDS\_NOWAKOWSKI\_SCIENCE\_2017\_NEN\_LATE\_UPREG |  | 360 | -0.29 | -1.17 | 0.091 | 0.214 | 1.000 | 1438 | tags=26%, list=15%, signal=30% |
| 80 | NB\_KI\_CLUSTER9 |  | 26 | -0.41 | -1.11 | 0.304 | 0.297 | 1.000 | 3529 | tags=50%, list=36%, signal=78% |
| 81 | VANGRONINGEN\_ADRN |  | 240 | -0.28 | -1.11 | 0.198 | 0.304 | 1.000 | 1368 | tags=23%, list=14%, signal=25% |
| 82 | ALMSTEDT\_17Q\_MSIGDB |  | 377 | -0.26 | -1.09 | 0.228 | 0.339 | 1.000 | 1478 | tags=17%, list=15%, signal=20% |
| 83 | ALMSTEDT\_11Q\_MSIGDB |  | 324 | -0.27 | -1.08 | 0.250 | 0.357 | 1.000 | 979 | tags=12%, list=10%, signal=13% |
| 84 | GANDAL\_ALCOHOLADDICTION\_UP\_PMID29439242 |  | 147 | -0.28 | -1.04 | 0.358 | 0.423 | 1.000 | 1232 | tags=18%, list=12%, signal=20% |
| 85 | RICHARDS\_NOWAKOWSKI\_SCIENCE\_2017\_MGE\_IPC2\_UPREG |  | 498 | -0.25 | -1.04 | 0.302 | 0.421 | 1.000 | 2235 | tags=28%, list=23%, signal=34% |
| 86 | PARADA\_T2\_DN\_26461091 |  | 48 | -0.33 | -1.03 | 0.391 | 0.437 | 1.000 | 1098 | tags=17%, list=11%, signal=19% |
| 87 | DOROSZKO\_U3180MG\_CLUSTER\_1 |  | 19 | -0.41 | -1.03 | 0.424 | 0.438 | 1.000 | 681 | tags=21%, list=7%, signal=23% |
| 88 | WANG\_PRONEURAL\_PMID\_28697342 |  | 16 | -0.42 | -1.03 | 0.423 | 0.437 | 1.000 | 382 | tags=25%, list=4%, signal=26% |
| 89 | DOROSZKO\_U3013MG\_CLUSTER\_1 |  | 38 | -0.34 | -1.02 | 0.414 | 0.439 | 1.000 | 1540 | tags=39%, list=16%, signal=47% |
| 90 | NB\_KI\_CLUSTER24 |  | 210 | -0.26 | -0.99 | 0.452 | 0.501 | 1.000 | 4419 | tags=52%, list=45%, signal=92% |
| 91 | GANDAL\_SCHIZOPHRENIA\_DN\_PMID29439242 |  | 17 | -0.40 | -0.99 | 0.463 | 0.497 | 1.000 | 1247 | tags=24%, list=13%, signal=27% |
| 92 | ROSEN\_KRONA\_TI\_UNPUBL |  | 59 | -0.30 | -0.97 | 0.481 | 0.542 | 1.000 | 485 | tags=10%, list=5%, signal=11% |
| 93 | GANDAL\_IBD\_UP\_PMID29439242 |  | 61 | -0.30 | -0.96 | 0.531 | 0.572 | 1.000 | 762 | tags=21%, list=8%, signal=23% |
| 94 | GAROFANO\_IAVARONE\_PPR\_SUBTYPE |  | 427 | -0.23 | -0.95 | 0.647 | 0.583 | 1.000 | 2748 | tags=32%, list=28%, signal=42% |
| 95 | NB\_KI\_CLUSTER20 |  | 23 | -0.29 | -0.79 | 0.775 | 0.904 | 1.000 | 269 | tags=9%, list=3%, signal=9% |
| 96 | NB\_KI\_CLUSTER15 |  | 187 | -0.16 | -0.62 | 1.000 | 0.994 | 1.000 | 2946 | tags=28%, list=30%, signal=40% |
| 97 | SEGERMAN\_MULTITHERAPY\_RESISTANCE\_FIG5A\_PMID\_27974212 |  | 32 | -0.19 | -0.53 | 0.994 | 0.996 | 1.000 | 2296 | tags=22%, list=23%, signal=28% |
Table: Gene sets enriched in phenotype **na **[plain text format]****
